# Effects of Wharton’s Jelly Mesenchymal Stem Cell-Derived Secretome on Cell Functions in Human Endometrium

**DOI:** 10.3390/cells15161504

**Published:** 2026-08-21

**Authors:** Silviya Doneva, Kalina Belemezova, Vesela Stoycheva, Ivan Bochev, Tanya Timeva, Maria Yunakova, Petya Andreeva, Katerina Kavaldzhieva, Atanas Shterev, Stanimir Kyurkchiev

**Affiliations:** 1 Tissue Bank BulGen, 1330 Sofia, Bulgaria; silvia@bulgen.com (S.D.); stoycheva.vesela@gmail.com (V.S.); stanimirkyurkchiev@gmail.com (S.K.); 2 Department of Biology, Medical Faculty, Medical University-Sofia, 1431 Sofia, Bulgaria; kkavaljieva@medfac.mu-sofia.bg; 3 Ob/Gyn Hospital Dr. Shterev, 1330 Sofia, Bulgaria; lakatush@yahoo.com (I.B.); ttimeva@yahoo.com (T.T.); m_yunakova@yahoo.com (M.Y.); andreivp3@gmail.com (P.A.); ashterev@gmail.com (A.S.); 4 Department of Molecular Immunology, Institute of Biology and Immunology of Reproduction, Bulgarian Academy of Sciences, 1113 Sofia, Bulgaria; 5 Department of Health Care, University of Ruse, 7017 Ruse, Bulgaria; 6 Department of Obstetrics and Gynecology, Medical Faculty, Medical University-Sofia, 1431 Sofia, Bulgaria; 7 Department of Health Care, Faculty of Public Health, Health Care and Sports, South-West University Neofit Rilski, 2700 Blagoevgrad, Bulgaria

**Keywords:** WJ-MSC secretome, endometrial stromal cells, proliferation, migration, angiogenesis

## Abstract

Background: Thin endometrium is a significant cause of infertility due to impaired regeneration, reduced receptivity, and insufficient angiogenesis. Mesenchymal stem cell-derived secretome (MSCsec) is a promising cell-free therapy because it contains bioactive molecules that promote tissue repair. This study evaluated the effects of Wharton’s jelly-derived MSC secretome (WJ-MSCsec) on human endometrial stromal cells (EnSCs) and endothelial cells in vitro. Methods: WJ-MSCs were isolated from umbilical cord tissue, characterized, and cultured under serum-free conditions. The concentrated secretome was analyzed using a human angiogenesis proteome array. EnSCs were isolated from endometrial biopsies. EnSC proliferation and migration in the presence of WJ-MSCsec were assessed using CCK-8 and scratch wound-healing assays. Pro-angiogenic activity of WJ-MSCsec was evaluated using a Matrigel tube formation assay with human umbilical vein endothelial cells (HUVECs). Results: Proteomic analysis of WJ-MSCsec revealed the presence of angiogenic, mitogenic, immunomodulatory, and chemotactic factors. Treatment with WJ-MSCsec significantly enhanced EnSC proliferation and accelerated wound closure. Furthermore, WJ-MSCsec markedly promoted endothelial tube formation, increasing total tube length, junction number, and mesh formation. Conclusions: WJ-MSCsec stimulates EnSC proliferation and migration while enhancing angiogenesis in vitro. WJ-MSCsec represents a promising cell-free therapeutic strategy for endometrial regeneration and reproductive disorders associated with impaired endometrial function.

## 1. Introduction

The International Society of Cellular Therapy (ISCT) defined mesenchymal stem cells (MSCs) as multipotent cells with the capacity for self-renewal and differentiation into osteoblasts, adipocytes, and chondroblasts in vitro. Additional characteristics of MSCs include plastic adherence and the expression of CD105, CD73, and CD90, alongside the absence of the markers CD45, CD34, CD14 or CD11b, CD79alpha or CD19, and HLA-DR [1]. Originally isolated from bone marrow, MSCs have since been identified in numerous tissues, including adipose tissue, skeletal muscle, skin, lung, Wharton’s jelly, placenta, endometrium, and menstrual blood. Although MSCs from different sources share a similar phenotype based on the aforementioned criteria, there are tissue-specific differences, especially in their growth characteristics and secretory profiles [2]. MSCs are involved in the regulation of various biological functions, including cell proliferation, activation, and apoptosis, and have a crucial role in tissue repair through their antifibrotic, anti-apoptotic, proangiogenic, and neuroprotective effects [3]. The molecular mechanisms by which MSCs exert their effects include both direct cell-to-cell interactions and paracrine effects from MSC-secreted products. All biologically active factors secreted by MSCs, including growth factors, cytokines, and extracellular vesicles (EV) like exosomes (40–120 nm) that contain and can transfer proteins and genetic material (such as microRNAs), are collectively referred to as the MSC-derived secretome. Numerous studies indicate that the MSC-derived secretome can have therapeutic effects independently of direct cell-to-cell contact [4,5].

The human endometrium undergoes cyclic renewal, which is controlled by reproductive hormones with the ultimate aim of preparing the uterus for embryo implantation and ensuring optimal conditions for pregnancy progression. Stromal, endothelial, epithelial, and immune cells form the cellular basis necessary for the optimal endometrial regeneration at monthly intervals. Adult stem cells, located either in the basal layer of the endometrium or recruited from the circulation, are primary participants in the regenerative process [6]. In addition to their role in physiological processes such as cyclic endometrial regeneration, stem cells have been proposed to contribute to several endometrial pathologies, including endometriosis, endometrial atrophy, and hyperplasia, which may be associated with abnormal proliferation of endometrial cells [7,8,9]. Thin endometrium (<7 mm thickness) is generally associated with lower rates of both natural conception and pregnancy following IVF/ICSI treatment of infertile patients. Various causes of this condition have been identified, including acute or chronic endometritis, repeated curettage, hysteroscopic procedures, and iatrogenic factors [10,11]. It is generally accepted that the primary mechanism underlying the condition of thin endometrium is the reduced responsiveness of the functional layer of the endometrium to hormonal stimulation, and that is the major cause for infertility. Another important aspect is that the endometrium is a site of active physiological angiogenesis, which is essential for the development of receptive decidua capable of accepting the implanting embryo. Any disruption or insufficiency in neoangiogenesis may therefore result in infertility due to impaired implantation [12].

Thin endometrium has been detected in approximately 2.4–5% of women under 40 years of age, while its prevalence may reach up to 25% in women over 40 years, according to some authors [13]. The treatment of thin endometrium remains challenging, as many different therapeutic approaches have been used, including hormone replacement therapy, administration of cytokines and growth factors (e.g., G-CSF), low-dose aspirin, and others. Recently, autologous platelet-rich plasma has been used in infertile patients with thin endometrium, showing some positive effects [14].

Over the past decades, several populations of cells with multipotent stem cell characteristics have been identified in the human endometrium [6,8,15], and these cells are largely engaged in endometrial regeneration. Research interest has been focused predominantly on multipotent mesenchymal stem/stromal cells (enMSCs) due to their capacity to undergo decidualization, a process that is essential for embryo implantation.

EnMSCs have been isolated and cultured from both the basal and functional layers of human endometrium, as well as from menstrual blood [16]. Following extensive characterization and in vitro expansion, these cells have been used for the treatment of some uterine pathologies such as thin endometrium [17], Asherman’s syndrome, POI (premature ovarian insufficiency) [18], and repeated implantation failure (RIF) [19]. In patients with POI, various types of mesenchymal stem cells have been used therapeutically, including bone marrow-derived MSCs [20], umbilical cord MSCs [21,22], and adipose tissue-derived MSCs [23].

The application of the MSC secretome as a therapeutic approach has certain advantages over transplantation of living stem cells. These include the absence of immune rejection risk or ectopic homing, and the presence of an abundance of growth factors, cytokines, and miRNA [24].

The aim of the present study was to evaluate the effects of Wharton’s jelly-derived MSC secretome (WJ-MSCsec) on the biological functions of cells in the human endometrium in vitro, as a prerequisite for subsequent clinical testing of the MSC-derived secretome.

## 2. Materials and Methods

### 2.1. Isolation and Culture of Human WJ-MSCs

Human mesenchymal stem cells were isolated from umbilical cord samples donated by healthy donors (37–40 gestational weeks) following written informed consent, as approved by the Executive Agency of Medical Supervision, Sofia, Bulgaria (Permit No. 5-00-1/18.07.2019).

Umbilical cord samples (15–20 cm) were collected immediately after delivery, placed in 50 mL tubes containing saline solution supplemented with 1% Penicillin/Streptomycin/Amphotericin B Mix (PAN Biotech, Aidenbach, Germany), and delivered to the laboratory. Under sterile conditions in a class II laminar flow cabinet (Thermo Scientific, Waltham, MA, USA), umbilical cord pieces were briefly rinsed with 70% ethanol (Sigma-Aldrich, Burlington, MA, USA) and washed extensively with sterile phosphate-buffered saline (PBS pH7.2, Sigma-Aldrich, Burlington, MA, USA). The umbilical cords were cut into 6–8 cm pieces and carefully dissected to expose and remove both umbilical arteries and the vein. The inner surface of the remaining tissue was placed face-down in a small volume of 0.25% collagenase I (*w*/*v*) (Genaxxon, Ulm, Germany) and 40 mg/mL hyaluronidase (Genaxxon, Ulm, Germany), ensuring that the outer surface was not exposed to enzymatic treatment. Samples were incubated for 45 min at 37 °C, after which loosened cells were gently scraped using a scalpel blade. The detached material was further processed with 10 mL of 0.25% collagenase I and 40 mg/mL hyaluronidase for an additional 45 min on a rotating shaker (SIA BioSan, Riga, Latvia) at 37 °C. Following digestion, 20 mL of sterile saline (B. Braun, Melsungen, Germany) was added, and the homogenized tissue was passed through a 70 µm cell strainer. The resulting cell suspension was centrifuged at 800× *g* for 10 min, and the cell pellet was resuspended in DMEM/F12 medium (PAN Biotech, Aidenbach, Germany) supplemented with 10% fetal bovine serum (FBS, PAN Biotech, Aidenbach, Germany), 10 ng/mL rHuFGF-β (Genaxxon, Ulm, Germany), and 1% Penicillin/Streptomycin/Amphotericin B Mix (PAN Biotech, Aidenbach, Germany). Cells were seeded at a concentration of 1 × 10^4^–4 × 10^4^ cells/cm^2^ in 25 cm^2^ culture flasks (Biologix, Camarillo, CA, USA) and incubated at 37 °C, 5% CO_2,_ and 95% humidity. After overnight incubation, non-adherent cells were removed, culture flasks were rinsed with sterile saline, and fresh medium was added. Medium was changed every 2–3 days, and cell growth was monitored using an inverted microscope (Leica, Wetzlar, Germany). At 80–90% confluence, cells were trypsinized and were either transferred into 75 cm^2^ flasks (Biologix, Camarillo, CA, USA) or cryopreserved in liquid nitrogen.

### 2.2. Characterization of WJ-MSCs

Surface marker expression of WJ-MSCs (passage 4) was analyzed by flow cytometry. Cells (concentration of 1 × 10^6^) were detached by trypsinization, washed in PBS (pH 7.2, Sigma-Aldrich, Burlington, MA, USA), and centrifuged at 800× *g* for 10 min. The cell pellet was additionally washed with 300 µL Cell Wash Solution (BD, San Jose, CA, USA). Afterwards, cells were incubated for 15 min in the dark with fluorochrome-conjugated specific antibodies against cell-surface markers (anti-CD45-FITC/CD34-PE, anti-CD73-PE, anti-CD90-FITC, anti-CD105-PerCP/Cy5-5, anti-CD44-FITC, anti-CD29-PE, anti-HLA-I-FITC) (all from eBioscience, San Diego, CA, USA). After washing, cells were fixed in 0.5 mL Cell Fix solution (BD, San Jose, CA, USA) and analyzed using a FACSCalibur (BD, San Jose, CA, USA) flow cytometer using BD CellQuest Pro software (BD, San Jose, CA, USA).

The differentiation potential of WJ-MSC (passage 5) was determined by induction of osteogenic and adipogenic differentiation. Adipogenic differentiation was induced using 1 μM dexamethasone (Sigma-Aldrich, Burlington, MA, USA), 5 μg/mL bovine insulin (Sigma-Aldrich, Burlington, MA, USA), 0.25 mM 3-isobutyl-1-methyl-xanthine (IBMX) (Sigma-Aldrich, Burlington, MA, USA), and 60 μM indomethacin (Sigma-Aldrich, Burlington, MA, USA). At the end of the induction period (21 days) cells were washed in PBS (pH 7.2, Sigma-Aldrich, Burlington, MA, USA) and fixed with 10% (*v*/*v*) neutral formalin (Merck, Darmstadt, Germany) for 30 min at room temperature, washed in distilled water, and stained in freshly prepared 0.6% Oil red O (Sigma-Aldrich, Burlington, MA, USA) solution for one hour. Characteristic staining of intercellular lipid droplets was observed under an inverted light microscope (Leica, Wetzlar, Germany) [25]. Osteogenic differentiation was induced for 21 days using 100 nM dexamethasone (Sigma-Aldrich, Burlington, MA, USA), 0.2 mM ascorbic acid-2-phosphate (Sigma-Aldrich, Burlington, MA, USA), and 10 mM β-glycerophosphate (Sigma-Aldrich, Burlington, MA, USA), and assessed by von Kossa and Alizarin Red staining (Sigma-Aldrich, Burlington, MA, USA) [25].

### 2.3. Collection of the WJ-MSC Derived Secretome

WJ-MSCs at the 4th passage were cultured to confluence, after which the medium was replaced with serum-free DMEM without phenol red or any supplements (PAN Biotech, Aidenbach, Germany) for 72 h. The conditioned medium was collected, centrifuged at 4000× *g* for 30 min to discard any cell debris, dead cells, and large apoptotic bodies, filtered through a 0.22 µm filter, and stored at −80 °C. Individual samples of WJ-MSC conditioned medium were pooled to overcome individual variability and used in functional experiments.

Secretome samples were concentrated using Amicon Ultra-15 centrifugal filters (3 kDa cut-off molecular weight) (Merck Millipore, Burlington, MA, USA) by centrifugation at 4000× *g* for 30 min. Protein concentration was determined by Bradford assay (Glentham Life Sciences, Corsham, UK) and turbidimetry (Cobas pro c 703, Roche, Basel, Switzerland; Cibalab, Sofia, Bulgaria); sterility was verified by hemoculture test (bioMérieux, Marcy-l’Étoile, France; Cibalab, Sofia, Bulgaria).

### 2.4. Analysis of the WJ-MSC Secretome Content

The WJ-MSC secretome was analyzed using the Proteome Profiler^TM^ Array Human XL Cytokine Array Kit (R&D Systems Inc., Minneapolis, MN, USA) following the manufacturer’s instructions. Briefly, secretome samples containing 200 µg/mL total protein were applied to the array membranes. Signal intensities were detected by chemiluminescence and quantified using ImageJ v.1.54g. Results for each factor were expressed as a percentage of the positive control signal.

### 2.5. Isolation, Culture, and Characteristics of Human Endometrial Stromal Cells (EnSCs)

Samples from human endometrium were collected by vacuum biopsy during the proliferative phase of the menstrual cycle following signed informed consent, which was approved by the Ethics Committee of Ob/Gyn Hospital Dr. Shterev, Sofia, Bulgaria (Protocol No. 2/02.04.2025). Endometrial tissue was briefly washed in 10% iodseptadone (Himax Pharma, Sofia, Bulgaria) and extensively rinsed in sterile phosphate-buffered saline (PBS, pH 7.2, Sigma-Aldrich, Burlington, MA, USA), minced and incubated in 10 mL enzymatic solution containing 0.25% collagenase I (*w*/*v*) (Genaxxon, Ulm, Germany) and 40 mg/mL (*w*/*v*) hyaluronidase (Genaxxon, Ulm, Germany), and incubated at 37 °C on a rotating shaker (SIA BioSan, Riga, Latvia) for 60 min. After enzymatic digestion, 20 mL of DMEM/F12 medium (PAN Biotech, Aidenbach, Germany) supplemented with 10% FBS (PAN Biotech, Aidenbach, Germany) and antibiotics/antimycotics (PAN Biotech, Aidenbach, Germany) was added, and the resulting tissue homogenate was passed through a 70 µm cell strainer (Greiner Bio-One, Kremsmünster, Austria) to obtain a single cell suspension, which was centrifuged at 800× *g* for 10 min. The pelleted cells were resuspended in complete DMEM/F12 (PAN Biotech, Aidenbach, Germany) with 10% FBS (PAN Biotech, Aidenbach, Germany), antibiotics/antimycotics (PAN Biotech, Aidenbach, Germany), rHuFGF-β (Genaxxon, Ulm, Germany), seeded in 6-well plate (Biologix, Camarillo, CA, USA) at a concentration of 2 × 10^4^ cells/cm^2^, and incubated at 37 °C, 5% CO_2_ and 95% humidity. Non-adherent cells were discarded, and fresh complete medium was added. Upon reaching approximately 80% confluence, cells were detached using Trypsin/EDTA (0.05%/0.02%) in DPBS (PAN Biotech, Aidenbach, Germany) and transferred into 25 cm^2^ flasks (Biologix, Camarillo, CA, USA) for continuous culture and further characterization [26].

To assess the functional properties of the isolated cells, human EnSCs (3rd passage) were cultured in DMEM (PAN Biotech, Aidenbach, Germany) supplemented with 10% charcoal-stripped fetal bovine serum (PAN Biotech, Aidenbach, Germany) until reaching 80–90% confluence. Cells were then washed with PBS (pH 7.2, Sigma-Aldrich, Burlington, MA, USA), and fresh medium containing 2% charcoal-treated FBS, 1.0 mmol/L 8-bromoadenosine-3′,5′-cyclic monophosphate (8-Br-cAMP; Sigma-Aldrich, Burlington, MA, USA) and 1.0 mmol/L synthetic progestin medroxyprogesterone acetate (MPA, Sigma-Aldrich, Burlington, MA, USA) was added. Culture supernatants were collected on days 3 and 5 and analyzed for prolactin concentration by chemiluminescence immunoassay on a Cobas 6000 analytical system (Roche Diagnostics, Rotkreuz, Switzerland; Cibalab, Sofia, Bulgaria).

Insulin-like growth factor binding protein 1 (IGFBP-1) concentrations in conditioned cell culture media were determined using a Human IGFBP-1 DuoSet ELISA (R&D Systems, Minneapolis, MN, USA) according to the manufacturer’s instructions. Recombinant IGFBP-1 standards ranging from 1.25 to 20 ng/mL were used to generate a standard curve. Samples and standards were analyzed in duplicate, and appropriate experimental controls were included. Absorbance was measured at 450 nm using an ELISA reader (Dynatech AG, Melville, NY, USA). IGFBP-1 concentrations were calculated from the standard curve using CurveExpert 1.34 software.

### 2.6. Proliferation Assays of Cultured Cells

Samples of tested cells (3 × 10^4^ cells per well/96-well plate) were cultured in the corresponding complete medium, either alone or supplemented with 20 µg/mL WJ-MSC secretome, for 7 days at 37 °C, 5% CO_2_. Subsequently, 10 µL/well of CCK-8 reagent (Sigma-Aldrich, Burlington, MA, USA) was added to each well. After incubation for 2 h at 37 °C, optical density (OD) was measured using an ELISA reader (BioTek 800TS, Winooski, VT, USA) at 450 nm.

### 2.7. In Vitro Wound Healing (Scratch) Assay

Human endometrial stromal cells (EnSCs) were cultured to confluence under standard conditions. A linear scratch was created across the cell monolayer using a sterile pipette tip, generating a cell-free area spanning across the whole field of view. Detached cells were removed by washing, and fresh medium was added.

Cells were treated as follows: untreated control and EnSCs treated with 20 µg/mL WJ-MSC secretome. Brightfield microscopic images were acquired at defined time points (0 h, ~37 h, ~61 h, and 72 h). Wound closure was quantified using an area-based method equivalent to automated segmentation in ImageJ v.1.54g. The open cell-free area between the left and right cellular fronts was defined as the wound region. Wound area percentage was plotted against time and calculated at ~37 h, ~61 h, and 72 h using ImageJ v.1.54g. Linear regression analysis over the first 72 h was used to calculate the wound closure rate, expressed as percentage of wound closure per hour (%/h). Comparative analyses were performed between control and treated groups.

### 2.8. Angiogenesis Assay

Human umbilical vein endothelial cells (HUVECs) (passage 4) were used to evaluate the effects of the WJ-MSC secretome. Pre-chilled flat-bottom 96-well microplates (SPL Life Sciences, Pocheon-si, Republic of Korea) were coated with 10 mg/mL Matrigel (Corning, Tewksbury, MA, USA) thawed overnight at 4 °C according to the manufacturer’s instructions. Plates were incubated for 60 min at 37 °C and 5%CO_2_ to allow gel polymerization. HUVECs were then seeded at a density of 1.0 × 10^4^/well in Endopan 3 medium (PAN Biotech Aidenbach, Germany). Cells were cultured under the following conditions: (A) Endopan 3 (control); (B) Endopan 3 + 50 ng/mL VEGF (positive control), and (C) Endopan 3 + 20 µg/mL WJ-MSC secretome. Formation of tubular networks was documented using an inverted microscope (Leica, Wetzlar, Germany) after 20 h incubation at 37 °C, 5% CO_2_. Total tube length, number of junctions, and number of meshes were quantified using Angiogenesis Analyzer for ImageJ v.1.54g [27].

### 2.9. Statistical Analysis

Statistical analyses were performed according to the number of groups compared. Data distribution was assessed using the Shapiro–Wilk test for normality, and homogeneity of variances was assessed using Levene’s test. Comparisons among multiple groups were performed using one-way analysis of variance (ANOVA) followed by the Holm–Šidák multiple comparisons test. Comparisons between two groups were performed using a two-tailed Student’s *t*-test with Welch correction, as appropriate. Statistical analyses were performed using GraphPad Prism version 8.0.1 (GraphPad Software, San Diego, CA, USA). Data are presented as mean ± standard deviation (SD). A *p*-value ≤ 0.05 was considered statistically significant.

## 3. Results

### 3.1. Assessment of the Stemness of WJ-MSCs

**Figure 1:**
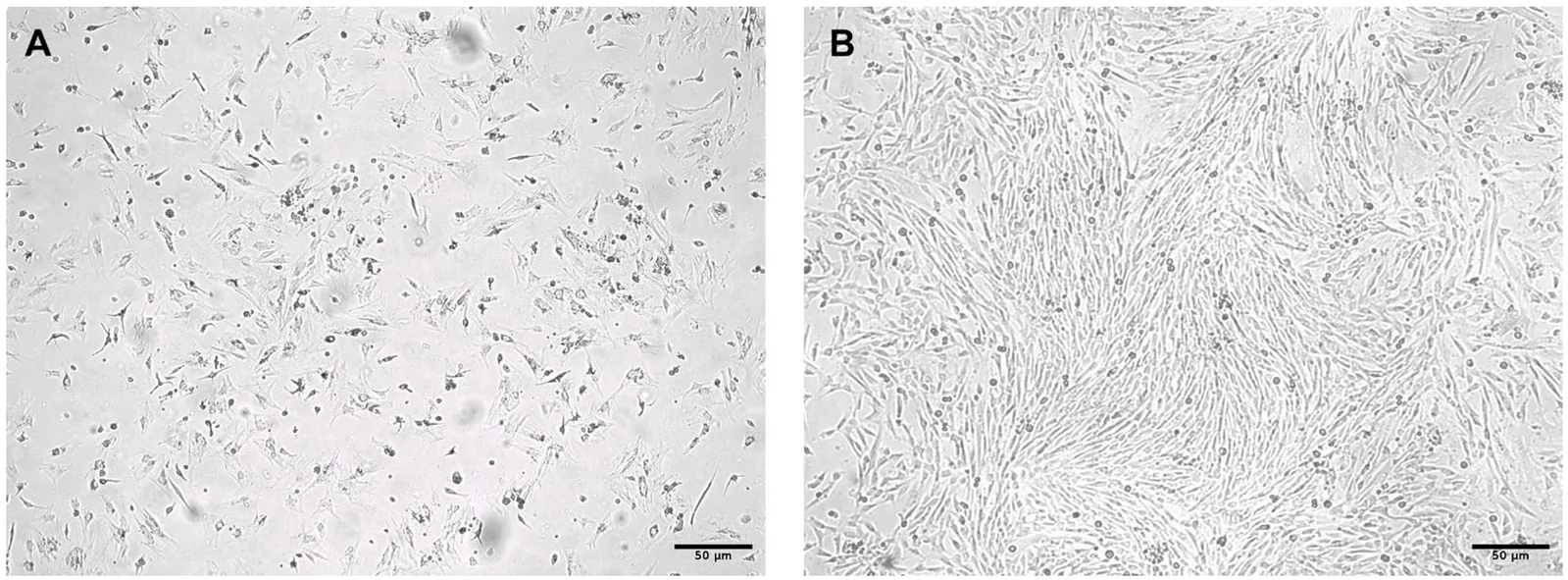
Representative light microscopy images of the morphological characteristics of isolated human WJ-MSCs. (**A**) Five days after initial culture (magnification, ×50); (**B**) confluent cell monolayer formed within 10 days (magnification, ×50).

Several days after initiation of cultures prepared from umbilical cord tissue (*n* = 10), elongated spindle-shaped cells with round nuclei were readily observed. These cells adhered to the plastic surface and formed expanding colonies that proliferated extensively, forming a homogeneous monolayer of fibroblast-like cells within 10–12 days (Figure 1A,B).

**Figure 2:**
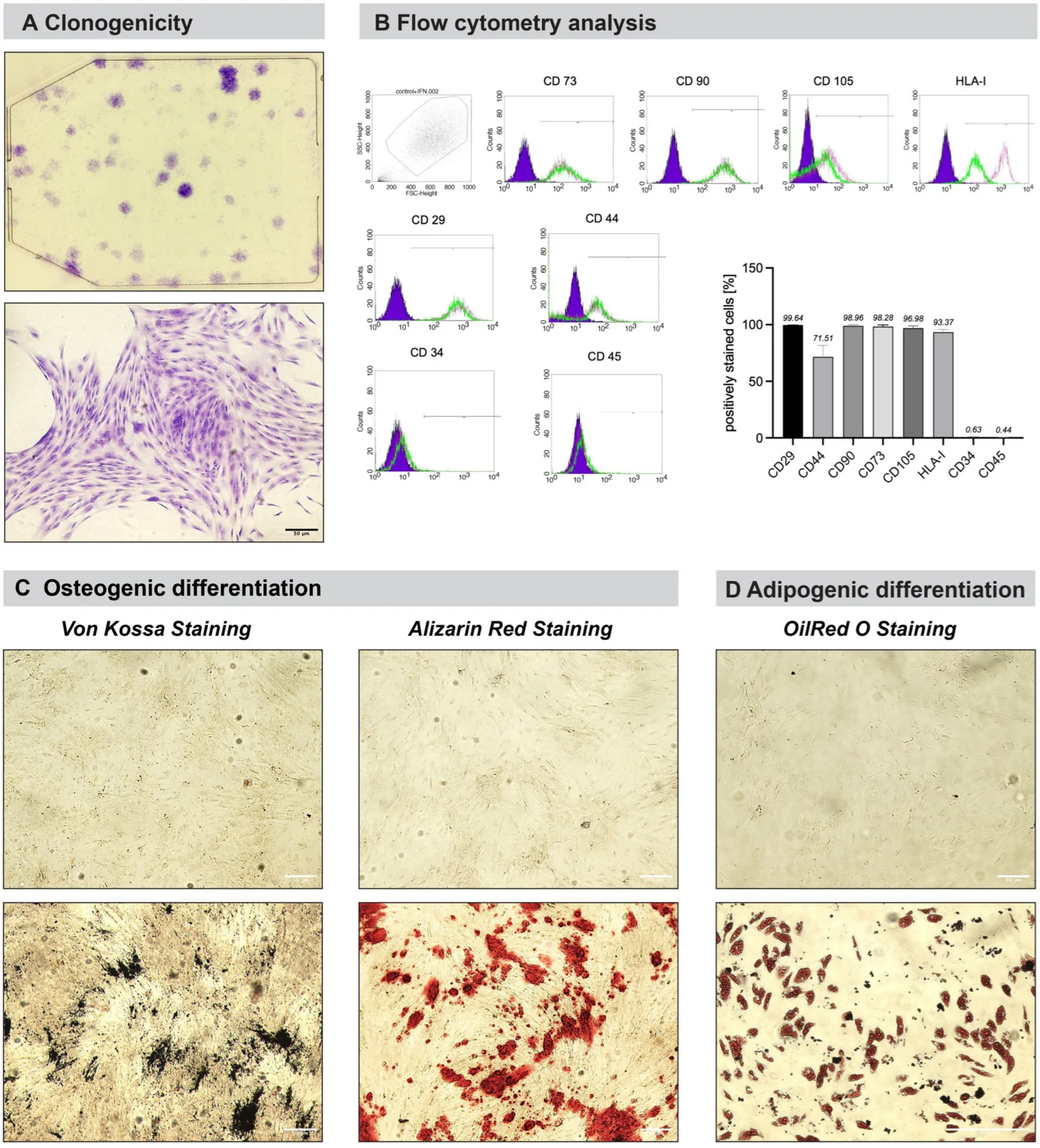
Characterization of WJ-MSCs. (**A**) Clonogenicity assay: representative images of stained with 0.5% Crystal Violet colony-forming units (**top**) and spindle-shaped adherent cells (**bottom**) demonstrating fibroblast-like morphology (magnification, ×50); (**B**) flow cytometry analysis: representative histograms showing the expression profile of surface markers. Cells display a typical mesenchymal phenotype. Quantification of marker expression is summarized in the bar graph below. Data are presented as mean ± standard deviation (SD), *n* = 10; (**C**) osteogenic differentiation: representative images of cells cultured under osteogenic conditions, showing mineralized matrix deposition as evidenced by positive staining with Von Kossa and Alizrain red, compared to undifferentiated controls (magnification, ×50); (**D**) adipogenic differentiation: Representative images of cells induced toward adipogenic differentiation, demonstrating intracellular lipid droplet accumulation visualized by positive red staining (OilRed O), compared to control conditions (magnification, ×100).

The isolated and cultured cells exhibited morphology consistent with mesenchymal stem cells according to the criteria of the International Society for Cellular Therapy [1]. To confirm stemness, a colony-forming assay was performed, which determines the self-renewal potential of MSCs by measuring the ability of a cell to clone itself and grow into a full colony. WJ-MSCs at passage 3 were seeded at a density of 10 cells/cm^2^ in 6-well plates and cultured for 10 days. Formed colonies were stained with 0.5% Crystal Violet (Merck, Germany) in methanol, and colony-forming unit-fibroblasts (CFU-F) consisting of at least 50 cells were counted on a stereomicroscope (Figure 2A).

Cells were further analyzed for surface antigen expression by fluorescence-activated cell sorting (FACS). More than 95% of cells were positive for CD73, CD90, CD105, CD44, CD29, and HLA-I, which are established markers of mesenchymal stem cells. In contrast, cells were negative for hematopoietic markers CD45 and CD34 (Figure 2B).

To further confirm isolated cells’ stemness, osteogenic differentiation was induced by culturing cells in the presence of osteoinductive factors as described in the Materials and Methods section. Successful osteogenic differentiation was demonstrated by deposition of dark-stained mineralized matrix following von Kossa staining. Increased alkaline phosphatase activity was also detected, confirming their potential for osteogenic differentiation (Figure 2C). Adipogenic differentiation capacity was demonstrated by the formation of red-stained triglyceride-containing vacuoles (Figure 2D).

The isolated cells were comprehensively characterized, as previously described in our studies, based on their morphological characteristics, clonogenic potential, multilineage differentiation capacity, and immunophenotypic profile (Figure 1 and Figure 2) [28,29]. The observed characteristics were consistent with the established criteria for the identification and characterization of mesenchymal stromal cells recommended by the International Society for Cellular Therapy (ISCT) [1]. Collectively, these results clearly prove that the isolated and cultured Wharton’s jelly-derived cells exhibit all defining characteristics of mesenchymal stem cells and are hereafter referred to as WJ-MSCs.

### 3.2. Analysis of the WJ-MSC Secretome by Proteome Profiler

**Figure 3:**
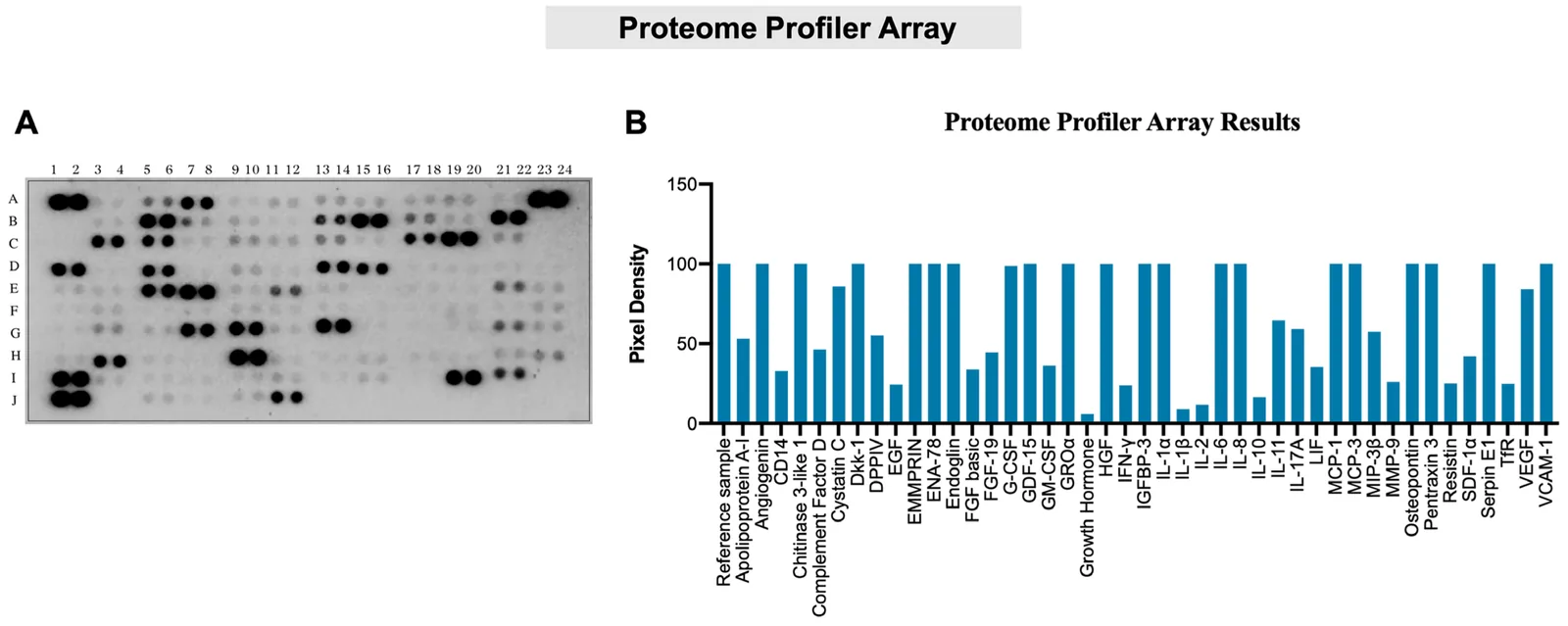
WJ-MSC secretome Proteome Profiler Array results analyzed by ImageJ software. (**A**) Assay membrane showing protein expression profiles. (**B**) Semi-quantitative densitometric analysis of array spot intensities performed using ImageJ v.1.54g. The secretome contained different biologically active proteins, including angiogenesis-related factors (Angiogenin, ENA-78/CXCL5, Endoglin, GDF-15, IL-1α, VEGF), mediators of leukocyte activation and migration (EMMPRIN, GROα, MCP-1/CCL2, MCP-3/CCL7, VCAM-1), immune modulators (IL-6, IL-8, IL-17A, Pentraxin 3), mitogenic and cell survival factors (basic FGF, Osteopontin), and growth factors (G-CSF, GM-CSF, HGF, VEGF). Cytokines typically produced by immune cells were not detected.

Following dialysis and lyophilization, the WJ-MSC-derived secretome was reconstituted in 1 mL PBS, adjusted to 200 µg/mL total protein, and analyzed using Proteome Profiler Array according to the manufacturer’s instructions. ImageJ analysis revealed positive signals for several biologically active factors, which were grouped as follows: (A) Angiogenesis-related factors: Angiogenin, ENA-78 (CXCL5), Endoglin, GDF-15, IL-1α, VEGF; (B) factors involved in leukocyte activation and migration: EMMPRIN, GROα, MCP-1 (CCL2), MCP-3 (CCL7), VCAM-1; (C) immune modulation: IL-6, IL-8, IL-17A, MCP-1 (CCL2), MCP-3 (CCL7), Pentraxin 3; (D) mitogenic and cell survival factors: FGF basic, Osteopontin; (E) growth factors: G-CSF, GM-CSF, HGF, VEGF. Notably, cytokines typically produced by immune cells were not detected, as expected (Figure 3). Detailed information regarding the Proteome Profiler coordinates is presented in Table 1.

For the treatment of cells in the subsequent experiments, a concentration of 20 μg/mL was selected based on a series of preliminary experiments in which different concentrations of the WJ-MSC secretome were evaluated using several experimental approaches. Among the concentrations tested, 20 μg/mL consistently produced the most optimal and reproducible biological responses and was therefore used for cell treatment.

### 3.3. Characterization of Human EnSCs

**Figure 4:**
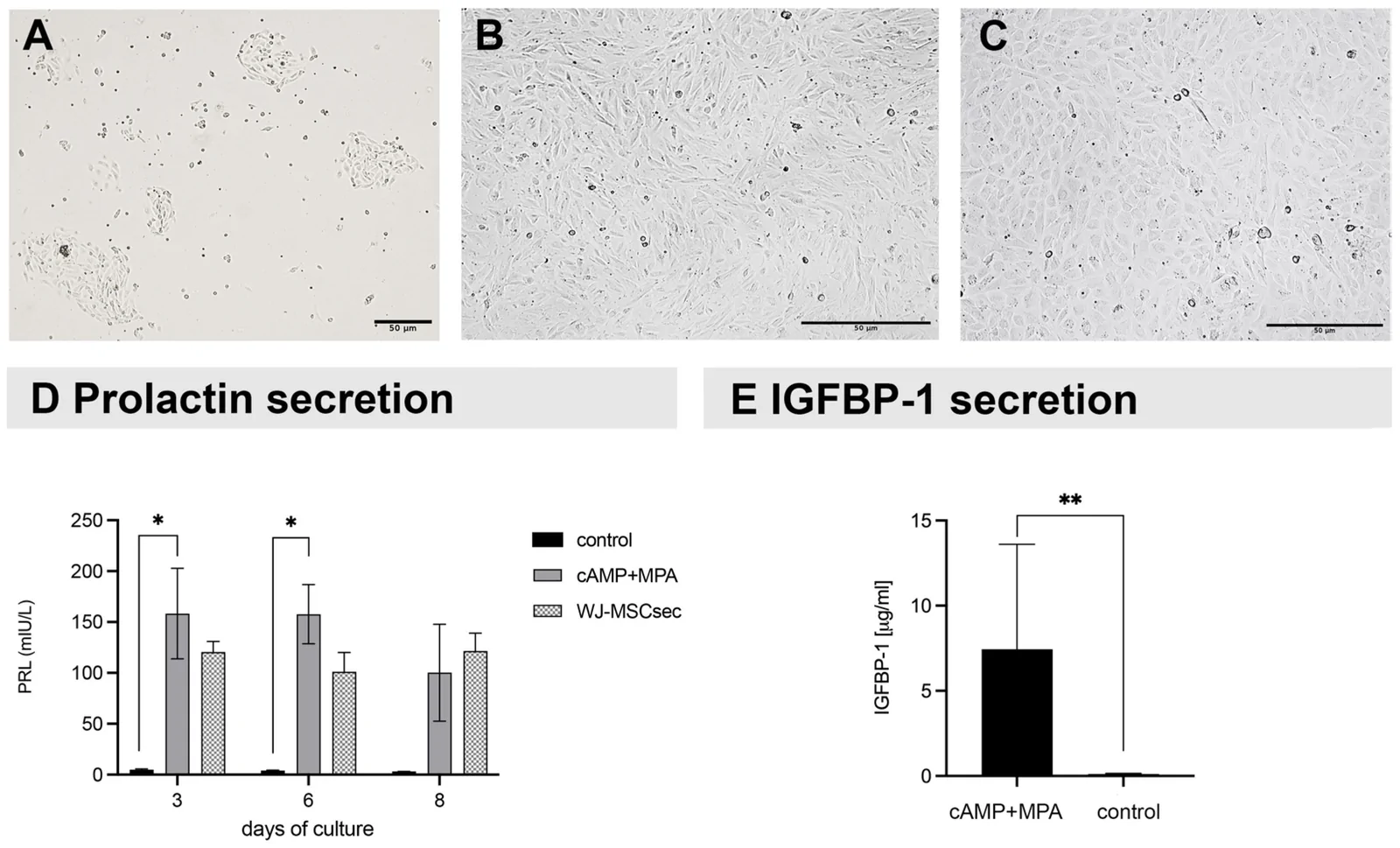
Endometrial stromal cells cultured in vitro: (**A**) Day 2, initial growth as “nests” of elongated cells (magnification, ×50); (**B**) Day 13, formation of a confluent monolayer (magnification, ×100); (**C**,**D**) decidualized EnSCs cultured in the presence of cAMP and MPA (magnification, ×100). On days 3 and 6, prolactin secretion from EnSCs cultured in the presence of cAMP and MPA was significantly higher than that of control cells. WJ-MSCsec did not have a significant effect on the prolactin secretion of EnSCs. (**E**) On day 6, IGFBP-1 concentration in culture media from EnSCs cultured in the presence of cAMP and MPA was also significantly higher than that of control cells (** *p* < 0.01). Bars represent mean ± SD (*n* = 5), * *p* < 0.05 was considered statistically significant.

Human endometrial stromal cells (EnSCs) were isolated from human endometrium (*n* = 5) obtained by vacuum pipelle biopsy and processed in the laboratory within 60 min after collection. Initially, isolated EnSCs grew as isolated “nests” consisting of elongated cells, which gradually expanded and formed a confluent monolayer. (Figure 4A,B). When exposed to decidualization-inducing factors (cAMP and progesterone), EnSCs underwent morphological changes, acquiring a flattened polygonal epithelioid-like appearance (Figure 4C), and secreted prolactin, which was detected in culture supernatants by a chemiluminescent assay. On day 3, the concentration of secreted prolactin in cultures treated with MPA and cAMP (158.33 ± 44.55 mIU/L) was significantly higher compared to control cells (4.87 ± 0.80 mIU/L), *p* < 0.05. On day 6, a similar difference was observed (157.67 ± 29.09 mIU/L vs. 4.03 ± 0.51 mIU/L), *p* < 0.05. The presence of WJ-MSCsec had no effect on the prolactin secretion (Figure 4D).

The identity of the cells was further confirmed by quantifying the concentration of insulin-like growth factor-binding protein 1 (IGFBP-1). On day 6, the concentration of IGFBP-1 was significantly higher in cells treated with cAMP and MPA (7.45 ± 6.16 µg/mL) compared with that in control cells (0.11 ± 0.03 µg/mL), *p* < 0.01 (Figure 4E).

The primary EnSCs used in the present study were characterized based on their typical morphological features and functional capacity to undergo decidualization, as demonstrated by the secretion of prolactin and IGFBP-1 (Figure 4). Taken together, the morphological characteristics and functional validation of decidualization provide sufficient characterization and confirmation of the identity of the EnSCs used in the present study and support their use in the subsequent experiments.

### 3.4. Effect of the WJ-MSC Secretome on the Migration (Scratch Test) and Proliferation of EnSCs

**Figure 5:**
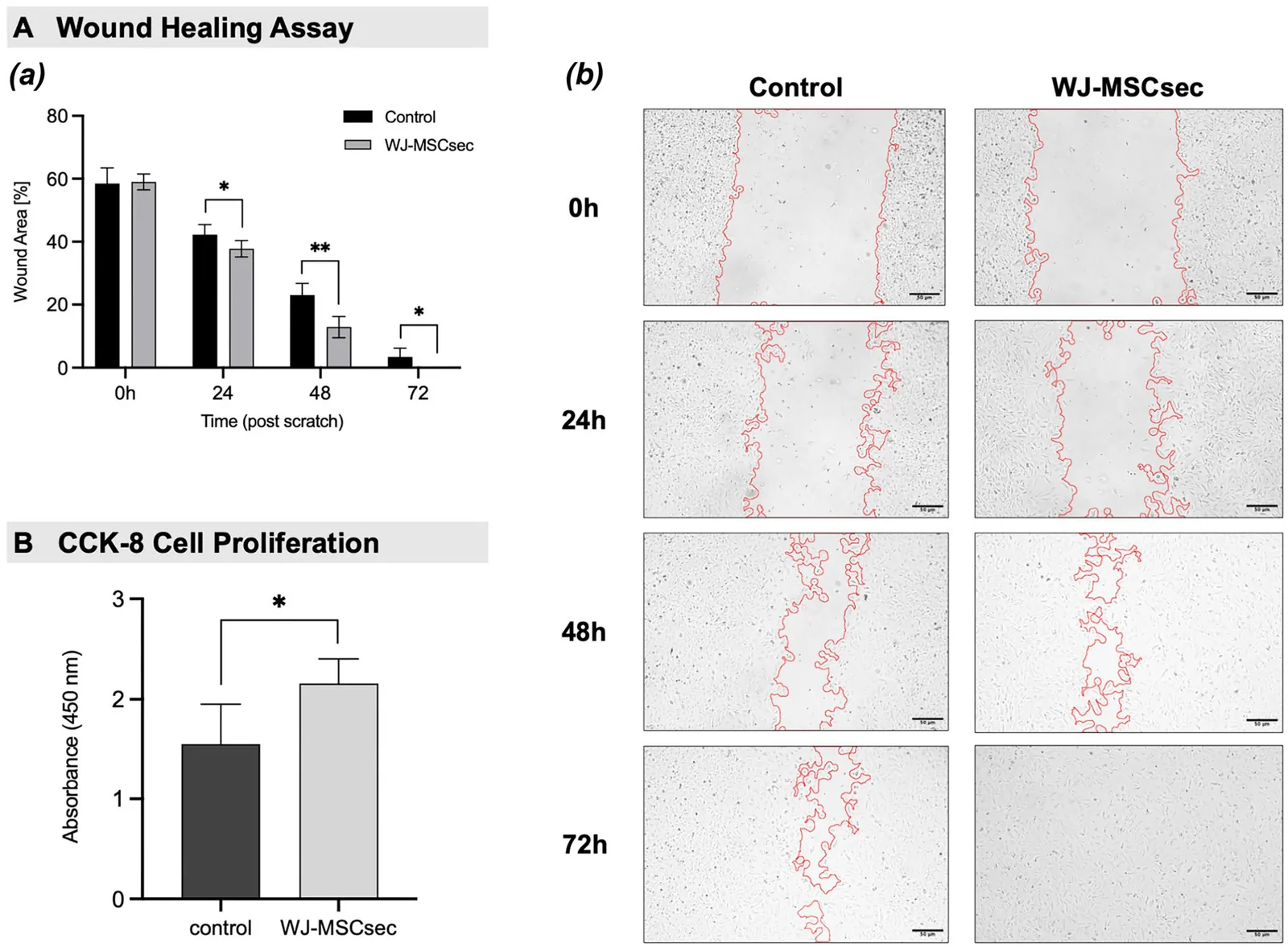
Effect of WJ-MSC secretome on the migration (Scratch Test) and proliferation of EnSCs. (**A**) Wound healing assay: (**a**) Analysis showed that WJ-MSC-derived secretome significantly accelerated EnSC wound closure compared with controls, with significant differences at 24 h (*p* < 0.05), 48 h (*p* < 0.01) and 72 h (*p* < 0.05). (**b**) Representative light-microscopy images showing EnSC migration into the scratch area at different time points following treatment with WJ-MSC secretome (magnification, ×50). Quantification of wound closure is performed using ImageJ v.1.54g. Data are expressed as mean ± SD. (**B**) EnSC proliferation (*n* = 5) was assessed using the CCK-8 assay after 7 days of culture in DMEM/F12 with 10% FBS (Control) or WJ-MSC secretome (20 µg/mL). EnSC proliferation was significantly increased following treatment with WJ-MSC secretome compared with the untreated control group (*p* < 0.05). Bars represent mean ± SD (*n* = 5), * *p* < 0.05 was considered statistically significant; ** *p* < 0.01.

Quantitative wound-healing analysis performed by ImageJ v.1.54g revealed a clear, time-dependent reduction in wound area under all experimental conditions, consistent with progressive migration of EnSCs into the scratched region. In control cultures (A), wound closure proceeded slowly (42.25 ± 3.15 at 24 h, 23.00 ± 3.78 at 48 h), with a substantial residual open area persisting at 72 h (3.41 ± 2.77) and complete closure achieved only at later time points (~96 h). In contrast, exposure to native WJ-MSC secretome (B) markedly accelerated wound closure (37.75 ± 2.61 at 24 h, *p* < 0.05, 12.88 ± 3.38 at 48 h, *p* < 0.01), resulting in complete coverage of the wound area by 72 h (Figure 5A). These findings indicate that secretome treatment robustly enhances the migratory capacity of human endometrial stromal cells, exerting the strongest pro-migratory effect during the early phase of wound healing.

Treatment with WJ-MSC secretome significantly increased EnSC proliferation. EnSC proliferation was evaluated on day 7 using the CCK-8 assay. EnSCs treated with WJ-MSC secretome (20 µg/mL) showed a significantly higher proliferation rate compared with control cells cultured in DMEM/F12 supplemented with 10% FBS alone (2.16 ± 0.25 vs. 1.55 ± 0.40, *p* < 0.05) (Figure 5B). These results suggest that WJ-MSC secretome contains bioactive factors that promote EnSC growth.

### 3.5. Effect of the WJ-MSC-Derived Secretome on the Function of HUVECs

**Figure 6:**
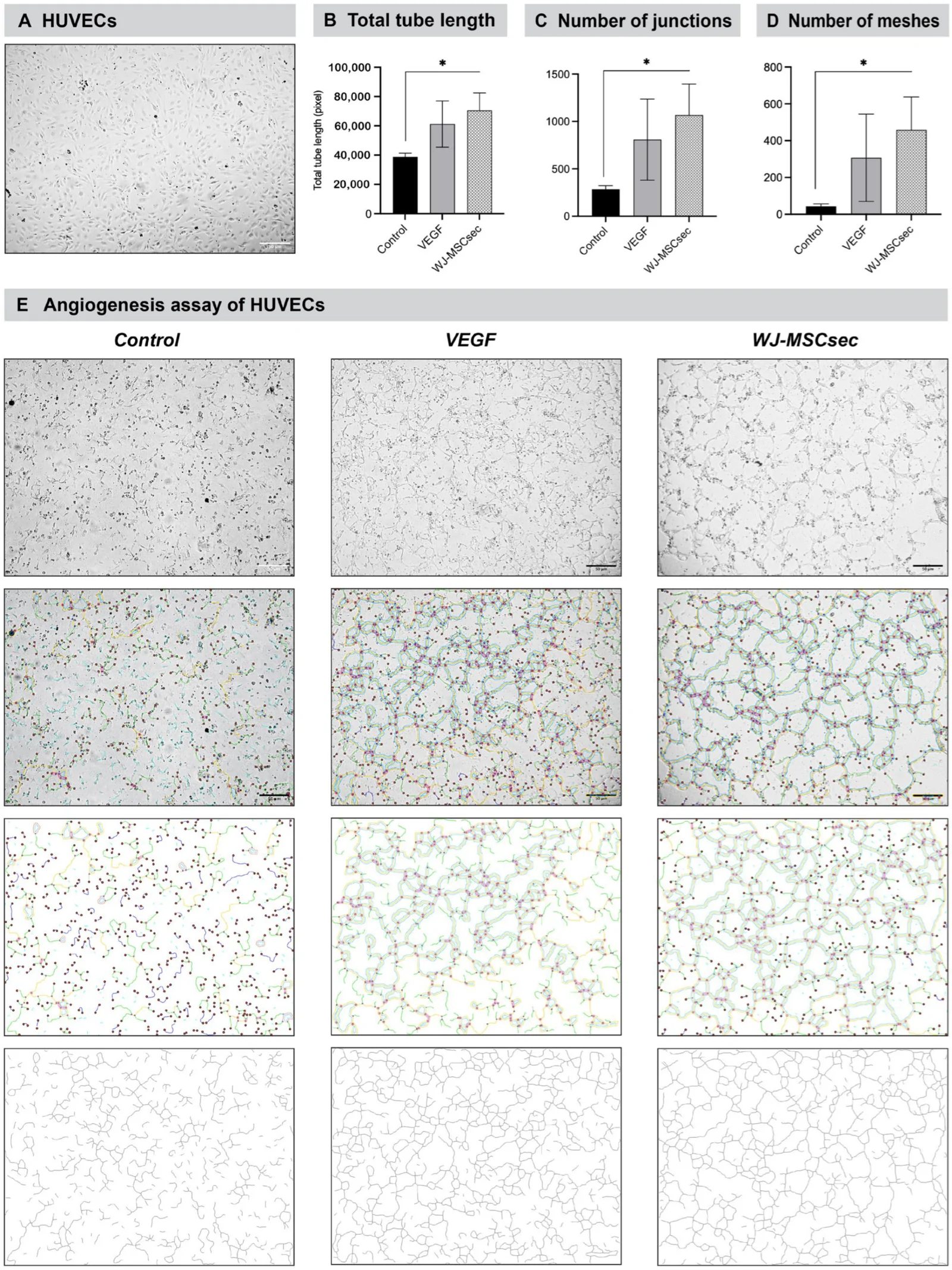
Angiogenesis tube-formation assay. (**A**) Representative light microscopy image of HUVECs culture at 90% confluence (magnification, ×50); capillary-like tube formation was assessed following 20 h of incubation with VEGF or WJ-MSCsec. Original images were analyzed using the Angiogenesis Analyzer for ImageJ, and the results for total tube length (**B**), number of junctions (**C**), and number of meshes (**D**) were presented. (**E**) Representative light microscopy images and maps of elements of HUVECs seeded on Matrigel in the presence of VEGF or 20 µg/mL WJ-MSCsec (magnification, ×50). Bars represent mean ± SD (*n* = 3), * *p* < 0.05 was considered statistically significant.

The tube formation assay (*n* = 3) demonstrated a clear pro-angiogenic effect of WJ-MSC secretome compared with control conditions (Figure 6). Quantitative analysis performed on ImageJ v.1.54g (Angiogenesis Analyzer) revealed a substantial increase in multiple parameters (total tube length, number of junctions, and number of meshes) associated with endothelial network formation. Secretome-treated cells exhibited a markedly higher total tubule length compared to control cells (70,553 ± 11,902 pixels vs. 38,844 ± 2507 pixels, *p* < 0.01), indicating enhanced endothelial elongation (Figure 6A). The WJ-MSC secretome increased the number of formed junctions and meshes compared to control cultures (1068 ± 326.8 vs. 286.1 ± 37.63 and 459 ± 178.5 vs. 43.64 ± 13.69, respectively, *p* < 0.01) (Figure 6C,D). Positive control cultures treated with VEGF only had a significant increase in all parameters compared to control cells (61,250 ± 15,826 pixels for total tube length, 807.9 ± 427.1 for number of junctions, and 307.3 ± 237.2 for number of meshes, *p* < 0.01) and were insignificant compared to WJ-MSCsec-treated cells. However, there was a clear tendency that WJ-MSCsec surpassed the VEGF positive control on all measured parameters of the angiogenesis assay.

## 4. Discussion

The present study demonstrates that the secretome derived from WJ-MSCs has pronounced biological effects on endothelial and endometrial stromal cells, which is highly relevant for the health of the female reproductive tract, supporting the concept that MSC-mediated therapeutic effects are largely driven by paracrine mechanisms rather than direct cell replacement [30]. Combining detailed characterization of WJ-MSCs, comprehensive profiling of their secreted factors, and functional assays on human endometrial stromal cells (EnSCs) and endothelial cells (HUVECs), our results provide mechanistic insight into how WJ-MSC secretome may contribute to tissue regeneration and functional remodeling of the endometrium.

The WJ-MSCs used in this study fulfilled the established criteria for mesenchymal stem cells, including plastic adherence, clonogenicity, multipotent differentiation potential, and a characteristic immunophenotype (CD73^+^, CD90^+^, CD105^+^, CD34^−^, CD45^−^). The use of freshly collected Wharton’s jelly tissue represents an important advantage, as it minimizes donor-related variability and age-associated functional decline observed in adult MSC populations. Furthermore, culturing these cells in serum-free medium ensured that the collected secretome predominantly reflected MSC-derived products rather than serum contaminants, thereby strengthening the biological relevance of downstream analyses.

Proteomic profiling revealed that the WJ-MSC secretome contains a broad spectrum of biologically active cytokines, chemokines, and growth factors, many of which are directly relevant to endometrial physiology. These factors can be functionally categorized into angiogenic mediators, regulators of immune cell recruitment and activation, modulators of inflammatory responses, and molecules involved in cell proliferation, migration, and survival. Importantly, classical immune cell-specific cytokines were not detected, consistent with the non-hematopoietic origin of WJ-MSCs and supporting the notion that their secretome promotes immune modulation rather than immune activation.

A detailed proteomic analysis of the MSC secretome was not the aim of our study. In these experiments, our objective was to prove the fundamental modulatory effects of the MSC secretome on key biological functions of endometrial stromal cells. Given the potential therapeutic application of MSC secretome in the treatment of thin endometrium, its pro-angiogenic capacity is of particular importance.

Angiogenesis is a critical process for cyclical endometrial regeneration, implantation, and placentation. The presence of potent angiogenic factors such as VEGF, angiogenin, endoglin, and GDF-15 in the WJ-MSC secretome provides a strong molecular basis for the enhanced tube formation observed in HUVEC assays. One study demonstrated that tissue origin can influence the MSC secretome protein content, with WJ-MSCsec retaining a better angiogenic profile when compared to other tissue sources [31]. VEGF, in particular, is a central regulator of endometrial vascular remodeling, while angiogenin and endoglin contribute to endothelial proliferation, migration, and vessel stabilization [32]. These findings suggest that WJ-MSC secretome likely creates a pro-angiogenic microenvironment supportive of endometrial repair and receptivity.

In addition to angiogenesis, stromal cell proliferation and migration are fundamental for endometrial regeneration during the menstrual cycle and following injury. Both bone marrow- and umbilical cord-derived MSC secretomes significantly increased EnSC motility, at least in part by upregulating CCL2 expression, suggesting a paracrine mechanism that supports endometrial regeneration [33]. Our results also demonstrated that WJ-MSC secretome significantly enhances EnSC proliferation and migratory activity, as evidenced by increased cell growth and accelerated wound closure in scratch assays. Quantitative, area-based analysis revealed a progressive reduction in wound size over time in all conditions, confirming active cell migration. These effects are likely mediated by a combination of mitogenic and survival-promoting factors identified in the secretome, including basic FGF, HGF, osteopontin, and G-CSF. Basic FGF and HGF are well-known regulators of stromal cell proliferation and motility, while osteopontin has been implicated in cell adhesion, migration, and endometrial receptivity. These findings indicate that secretome-derived factors may enhance the migratory response of EnSCs, suggesting a pro-regenerative effect on stromal cell dynamics.

HUVEC angiogenesis assay further supported these findings. WJ-MSC secretome-treated HUVECs formed a high-density, complex, and stable tubular network, whereas control cells displayed a medium-to-low density, more scattered, and less organized network structure. The increased numbers of junction meshes in the WJ-MSC secretome-treated group indicated improved structural integrity and stabilization of the endothelial network, hallmarks of functional angiogenesis. Other studies have also reported that the MSC secretome significantly enhanced endothelial cell proliferation, migration, and angiogenic capacity under high-glucose conditions, with the umbilical cord MSC secretome demonstrating the strongest reparative effects, particularly in promoting cell migration and tube formation [34,35]. The presence of potent angiogenic factors such as VEGF, angiogenin, endoglin, and GDF-15 in the WJ-MSC secretome provides a strong molecular basis for the enhanced tube formation observed in HUVEC assays. These findings suggest that the WJ-MSC secretome can create a pro-angiogenic microenvironment supportive of endometrial repair and receptivity.

Nevertheless, several limitations of the current study should be noted. All experiments were conducted in vitro, which cannot fully mirror the complex cellular interactions and microenvironmental signaling in the human endometrium. Although proteomic analysis identified numerous bioactive mediators, further studies should identify the specific mechanisms underlying the observed biological effects. Finally, the effects of exosomes, which are a major component of the MSC secretome, were not investigated separately.

Future studies should validate these findings in animal models and clinical trials to determine whether the regenerative effects of WJ-MSCsec can be translated into improved endometrial repair, vascularization, implantation rates, and fertility outcomes. Further investigation is needed to optimize and standardize secretome manufacturing, which is essential for advancing WJ-MSCsec toward clinical application as a cell-free therapy for endometrial regeneration.

## 5. Conclusions

Collectively, these findings support the hypothesis that WJ-MSC secretome may act as a bioactive cocktail capable of coordinating angiogenesis, stromal cell expansion, migration, and immune modulation—key processes underlying endometrial regeneration. From a translational perspective, the use of cell-free MSC-derived products offers several advantages over cell-based therapies, including reduced immunogenicity, easier standardization, and improved safety profiles. The demonstrated effects of WJ-MSC secretome on EnSCs and endothelial cells underscore its potential as a therapeutic tool for enhancing endometrial repair and improving reproductive outcomes.

## Figures and Tables

**Table 1:** Human XL Cytokine Array coordinates used for WJ-MSC secretome Proteome Profiler Array.

Coordinate	Analyte/Control	Coordinate	Analyte/Control	Coordinate	Analyte/Control
A1, A2	Reference Spots	D11, D12	IGFBP-2	G13, G14	MIF
A3, A4	Adiponectin	D13, D14	IGFBP-3	G15, G16	MIG
A5, A6	Apolipoprotein	D15, D16	IL-1α	G17, G18	MIP-1α/MIP-1β
A7, A8	Angiogenin	D17, D18	IL-1β	G19, G20	MIP-3α
A9, A10	Angiopoietin-1	D19, D20	IL-1ra	G21, G22	MIP-3β
A11, A12	Angiopoietin-2	D21, D22	IL-2	G23, G24	MMP-9
A13, A14	BAFF 10673	D23, D24	IL-3	H1, H2	Myeloperoxidase
A15, A16	BDNF 627	E1, E2	IL-4	H3, H4	Osteopontin
A17, A18	Complement Component C5/C5a	E3, E4	IL-5	H5, H6	PDGF-AA
A19, A20	CD14	E5, E6	IL-6	H7, H8	PDGF-AB/BB
A21, A22	CD30	E7, E8	IL-8	H9, H10	Pentraxin 3
A23, A24	Reference Spots	E9, E10	IL-10	H11, H12	PF4
B3, B4	CD40 ligand	E11, E12	IL-11	H13, H14	RAGE
B5, B6	Chitinase 3-like 1	E13, E14	IL-12 p70	H15, H16	RANTES
B7, B8	Complement Factor D	E15, E16	IL-13	H17, H18	RBP-4
B9, B10	C-Reactive Protein	E17, E18	IL-15	H19, H20	Relaxin-2
B11, B12	Cripto-1	E19, E20	IL-16	H21, H22	Resistin
B13, B14	Cystatin C	E21, E22	IL-17A	H23, H24	SDF-1α
B15, B16	Dkk-1	E23, E24	IL-18 Bpa	I1, I2	Serpin E1
B17, B18	DPPIV	F1, F2	IL-19	I3, I4	SHBG
B19, B20	EGF	F3, F4	IL-22	I5, I6	ST2
B21, B22	EMMPRIN	F5, F6	IL-23	I7, I8	TARC
C3, C4	ENA-78	F7, F8	IL-24	I9, I10	TFF3
C5, C6	Endoglin	F9, F10	IL-27	I11, I12	TfR
C7, C8	Fas Ligand	F11, F12	IL-31	I13, I14	TGF-α
C9, C10	FGF basic	F13, F14	IL-32	I15, I16	Thrombospondin-1
C11, C12	FGF-7	F15, F16	IL-33	I17, I18	TNF-α
C13, C14	FGF-19	F17, F18	IL-34	I19, I20	uPAR
C15, C16	Flt-3 Ligand	F19, F20	IP-10	I21, I22	VEGF
C17, C18	G-CSF	F21, F22	I-TAC	J1, J2	Reference Spots
C19, C20	GDF-15	F23, F24	Kallikrein 3	J5, J6	Vitamin D BP
C21, C22	GM-CSF	G1, G2	Leptin	J7, J8	CD31
D1, D2	GROα	G3, G4	LIF	J9, J10	TIM-3
D3, D4	Growth Hormone	G5, G6	Lipocalin-2	J11, J12	VCAM-1
D5, D6	HGF	G7, G8	MCP-1	J23, J24	Negative Controls
D7, D8	ICAM-1	G9, G10	MCP-3		
D9, D10	IFN-γ	G11, G12	M-CSF		

## Data Availability

The original contributions presented in this study are included in the article. Further inquiries can be directed to the corresponding author.

## Abbreviations

The following abbreviations are used in this manuscript:

ISCT	International Society of Cellular Therapy
MSCs	Mesenchymal Stem Cells
WJ-MSCs	Wharton’s jelly-derived MSC
EnMSCs	Endometrial Mesenchymal Stem Cells
POI	Premature Ovarian Insufficiency
RIF	Repeated Implantation Failure
WJ-MSCsec	Wharton’s jelly-derived MSC secretome
HUVEC	Human umbilical vein endothelial cells
VEGF	Vascular Endothelial Growth Factor
EV	Extracellular Vesicles
IVF	In vitro fertilization
ICSI	Intracytoplasmic Sperm Injection
PBS	phosphate-buffered saline
CCK-8	Cell Counting Kit-8
FACS	fluorescence-activated cell sorting
IGFBP-1	Insulin-like growth factor binding protein 1
